# Peer-led BASICS intervention to reduce alcohol consumption and alcohol-related consequences among university students: a randomized controlled trial

**DOI:** 10.3389/fpubh.2023.1280840

**Published:** 2023-10-31

**Authors:** María Lavilla-Gracia, María Pueyo-Garrigues, Diego Calavia Gil, Nuria Esandi-Larramendi, Cristina Alfaro-Diaz, Navidad Canga-Armayor

**Affiliations:** ^1^Department of Community, Maternity and Pediatric Nursing, School of Nursing, University of Navarra, Pamplona, Spain; ^2^Navarra Institute for Health Research (IdiSNA), Pamplona, Spain; ^3^Department of Otorhinolaryngology, Clinica University of Navarra, Pamplona, Spain; ^4^Department of Nursing Care for Adult Patients, School of Nursing, University of Navarra, Pamplona, Spain

**Keywords:** alcohol, university students, harm reduction, peer-led interventions, nursing students

## Abstract

**Introduction:**

Alcohol consumption is the main substance abused during university and is associated with physical, legal, emotional, social, and cognitive consequences. The peer-led BASICS intervention has been shown to be effective in decreasing the quantity and frequency of drinking, the estimated peak blood alcohol concentration (BAC), and the number of binge drinking episodes among this population.

**Objective:**

This study evaluated the effectiveness of the peer-led BASICS intervention to reduce risky alcohol consumption among university students in the Spanish context.

**Materials and methods:**

A two-arm randomized controlled trial in a university in northern Spain including 308 first- and second-year university students recruited between October 2022 to March 2023. The intervention was a 30-min in-person peer-led motivational interview. Participants were assessed at baseline and 1-month postintervention. The primary outcome was the quantity and frequency of alcohol consumption during a typical week. The intervention effect was verified using a mixed factorial ANOVA model.

**Results:**

Compared with students in the control group, students who received the intervention reduced the number of drinks per week by 5.7 (95% CI 5.54, 5.86); the number of drinks consumed in a typical weekend by 5.2 (95% CI 5.07, 5.33); the number of drinks consumed on the occasion of greatest consumption by 4.9 (95% CI 4.78, 5.02); the number of binge drinking episodes by 1.4 (95% CI 1.37, 1.43); the peak BAC on a typical week and on the occasion of greatest consumption decreased by 0.06 (95% CI 0.058, 0.062) and 0.09 (95% CI 0.088, 0.092); the number of alcohol-related consequences by 5.8 (95% CI 5.67, 5.93); and the motivation to change their alcohol use increased by −0.8 (95% CI −0.85, −0.75).

**Conclusion:**

The peer-led BASICS intervention is effective in changing alcohol consumption and its related consequences among Spanish university students in the short term. The action of nursing students as counselors positively impacted drinking patterns among their peers.

**Clinical trial registration:**

https://clinicaltrials.gov/study/NCT05639374?intr=Effectiveness%20of%20a%20Peer-led%20Program%20to%20Prevent%20Alcohol%20Consumption&rank=1&page=1&limit=10, identifier: NCT05639374.

## 1. Introduction

Alcohol consumption peaks in emerging adulthood (18–25 years old), a vital stage in which half of this population consume excessive alcohol or engage in binge drinking (five or more drinks on an occasion for men, or four or more drinks on an occasion for women) (1). Although the worldwide prevalence of binge drinking decreased from 22.6% in 2000 to 18.2% in 2016, the prevalence remains high (33.9%) in Europe (2). Specifically, hazardous drinking is the primary substance abuse problem during the university period. On average, students experience three episodes of abusive drinking per month, suffering physical, legal, emotional, social, and cognitive consequences with 10% developing alcohol dependence (3–5).

Motivational interventions (MI) have been shown to be effective when implemented in university settings (6). The program Brief Alcohol Screening and Intervention for College Students (BASICS), designed for college students, has been demonstrated to reduce alcohol use and related consequences in this population (6–8). It involves <2 h of direct contact and is delivered in the style of non-confrontational and non-judgmental motivational interviews, which supports individual student autonomy in setting goals for drinking and harm reduction planning (8, 9). Motivation for change is evoked through discussion of personalized feedback addressing students' alcohol use, consequences, beliefs about alcohol, and other contextual factors (9).

A recent literature review indicated that when BASICS is delivered by peers, there is a reduction in the quantity and frequency of drinking, estimated peak blood alcohol concentration (BAC) and number of binge drinking episodes among college students (10). Including peers as facilitators is becoming a viable strategy among universities to reduce harmful behaviors due to their great credibility (11). In addition, peer providers are considered competent and well-trained by university students (12, 13). Most peer-led BASICS interventions have taken place in the American context. In Spain, only one pilot study has assessed the preliminary efficacy and feasibility of a peer-led alcohol intervention to reduce alcohol consumption in university students (13). The findings suggested that the intervention may be effective for decreasing alcohol consumption and alcohol-related consequences in Spanish undergraduates (13). The current study follows up on previous findings using a large-scale controlled trial. The objective of this study was to evaluate the effectiveness of the peer-led BASICS intervention to reduce risky alcohol consumption among university students in the Spanish context. The main hypothesis was that the intervention would positively impact alcohol use by significantly decreasing the number of drinks consumed in a typical week.

## 2. Materials and methods

### 2.1. Study design

This was a two-arm randomized controlled trial conducted in a university in northern Spain. The trial protocol was registered with clinicaltrials.gov (NCT05639374).

### 2.2. Training of peer facilitators

The intervention was delivered by volunteer fourth-year undergraduate nursing students who attended a 12-week, 2 h training course (12 h for theoretical classes and 10 h for practice workshops) that was conducted over one semester, from September to December 2022. The course covered concepts related to MI that were structured into three modules: 1. motivational interviewing; 2. alcohol and university students; and 3. feedback (as a strategy for MI). Lectures were delivered by a faculty nurse member who was an expert in the field. The teaching methodologies were didactic lectures, video visualizations, and role play exercises. Upon completion of the theoretical classes, students conducted two *in vivo* simulations and motivational interviews with peers (undergraduates pursuing other degrees), which were videotaped. To maintain the fidelity of the intervention, all students were given tailored individual written feedback noting their strengths and areas for improvement. To provide this feedback, the videotaped *in vivo* simulations were coded using an *ad hoc* alcohol-related content checklist and the Peer Proficiency Assessment (14) as a tool for identifying MI-related behaviors and counting microskills. One session involving group supervision was also conducted to emphasize ways to improve the use of motivational interviewing skills. Only those students who were competent in motivational interviewing and that adhered to the intervention participated in the study.

### 2.3. Sample size

The sample size was estimated by assuming an expected decrease in quantity and frequency of alcohol consumption that was higher than that reported in a previous randomized controlled trial (15). We assumed a reduction of 4.81 points in the Daily Drinking Questionnaire in the intervention group. According to these assumptions, the required sample size to achieve a statistical power of 0.80 for analysis was 306 participants (153 per condition).

### 2.4. Participants and recruitment

The study participants were university students recruited from a university in northern Spain between October 1, 2022, and March 14, 2023. The inclusion criteria were as follows: age 18-years or older; first- or second-year student status; and currently engaging in binge drinking.

The recruitment strategy involved advertising in university classrooms and student residences and disseminating the study through the university's social networks and the student newsletter. One member of the research team provided information about the project and invited students to participate. It was explained to the participants that it was a health promotion project at the university with the objective of raising their awareness about their pattern of alcohol consumption and its effects on young people. They were blinded to the goal of the study (to reduce alcohol consumption) to prevent them from reporting lower alcohol use at the end of the study. Furthermore, they were not informed of the existence of two groups. Students signed up using a QR code redirected to a Google sheet to provide their name and contact email. Subsequently, they were e-mailed a document containing information about the study and the informed consent form that they had to return signed to participate in the study. Those students who met eligibility criteria were then randomly assigned to either the control or intervention group.

### 2.5. Randomization, allocation concealment, and blinding

Simple randomization of participants was performed by generating a random number sequence using Stata v.15. The number sequence was stored on an encrypted electronic file, and one researcher was the only study staff member with access to this information. Participants, following the order of enrolment in the study, were assigned to an intervention or control group according to the corresponding random sequence number. Study investigators, peer facilitators and participants were blinded to participant allocation.

### 2.6. Measures

The primary outcome was the quantity and frequency of alcohol consumption during a typical week, which was measured using the Daily Drinking Questionnaire-Revised (DDQ) (16). To facilitate its completion by the participants, they were asked by the specific number of different beverages they drank per week (for example, glasses of wine, champagne, cava, beer, cider, vermouth, shots, mixed drinks, fruit liqueurs or strong liquors). The researchers then transformed it into standard drink units.

The following eight secondary outcomes were measured: the number of drinks consumed on a typical weekend and on the occasion of greatest consumption, as measured through the DDQ; the highest BAC during a typical week and on the occasion of greatest consumption, estimated using the quantity/frequency/peak index (QF) Index (9); the frequency of binge drinking episodes, as measured using a closed-ended question [“during the last month, how many days did you consume five or more (for males)/four or more (for females) alcoholic beverages on the same drinking occasion?”]; the motivation to make a change in their alcohol consumption and self-efficacy, as assessed through a series of questions using a Likert scale from 1 to 10 (“How important is it for you to make any changes in your alcohol consumption?” and “How confident are you that you are capable of making any changes in your personal alcohol consumption?”); and the number of alcohol-related consequences, as measured using the Spanish version of the Young Adult Alcohol Consequences Questionnaire (S-YAACQ) (17). The S-YAACQ asked to participants to indicate whether they had experienced any of the 48 consequences of alcohol consumption with a dichotomous response format (yes/no). It is composed of eight subscales that are: self-perception, socio-interpersonal relationships, academic/work development, risk behaviors, memory loss due to alcohol consumption, deterioration of control, psychological dependence and self-care. The total score reflects the total number of consequences the individual experienced in the last month. This questionnaire has good internal consistency (Cronbach's alpha = 0.91) (18, 19) and for each of its dimensions (Cronbach's alpha values between 0.70 and 0.91) (17).

All outcome measures accounted for alcohol consumption during the past month.

These assessments were made twice: before (baseline assessment) and 1 month after the program (follow-up assessment).

### 2.7. Data collection

Participants were informed about their group assignment (intervention or control) by e-mail and were asked to complete the online baseline assessment using the Survey Monkey platform. This questionnaire included 25 questions divided into two sections: sociodemographic variables (first eight questions), such as sex, hometown, and faculty; and 17 questions about their history of alcohol consumption and alcohol-related consequences during the previous month. One month after the interview (for de intervention group) or after the baseline questionnaire (for the control group), students received an e-mail directing them to complete the 1-month follow-up assessment via Survey Monkey. This assessment consisted of the same alcohol-related questions referring to the outcome variables. If they did not complete it, they were sent a reminder and the link by the smartphone messaging app WhatsApp.

### 2.8. Intervention

The intervention is based on the BASICS manual (9) and on motivational interviewing theory (20). Participants received the peer-led BASICS intervention that consisted of a 30-min in-person motivational interview (MI). A peer counselor gave personalized graphical feedback including the following themes: (1) drinking pattern (e.g., quantity and frequency of alcohol consumption in a typical week, drinks consumed on the occasion of greatest consumption and binge drinking episodes); (2) blood alcohol concentration (BAC) reached (e.g., the student's BAC during a typical week, on the occasion of greatest consumption, and number of hours it takes for it to return to a zero BAC); (3) social norm of alcohol consumption (e.g., perception of the number of beverages consumed by their peers, perception of the number of university students who abstain and binge drink); (4) alcohol-related consequences (e.g., on a personal, social, academic or environmental level); (5) individual risk factors (e.g., age of onset of alcohol consumption, tolerance level, polydrug use and other drugs); (6) alcohol caloric consumption and hours of exercise required to burn those calories; (7) financial costs (e.g., approximate weekly and annual cost); (8) protective behavioral strategies; and (9) educational information. The intervention was held in two prepared rooms at the university (one in the bio-sanitary sciences building and the other in the social sciences building) to facilitate the attendance of the participants. The interventions were conducted from January to April 2023. During the study, the intervention was not modified.

Participants in the control group did not receive any specific intervention which is the standard-of-care at the university where the study took place.

### 2.9. Statistical analysis

Baseline data were reported as the mean [standard deviation (SD)] for continuous variables since all of them followed a normal distribution. Categorical variables were reported as percentages (*n*, %). Differences between the groups were evaluated using Student's *t* test to compare means and Pearson's χ^2^ test to compare proportions.

The data were analyzed on an intention-to-treat basis. The intervention effect was verified using a mixed factorial ANOVA model in which the intervention time (pre and post) was the within-subjects factor, and the intervention group (control and intervention) was the between-subjects factor. For statistically significant interactions, the differences between the groups at an intervention time were verified by means of contrasts using Fisher's least significant difference (LSD) adjustment. Normality was assessed using the Shapiro–Wilk test on the residuals together with visual verifications of the boxplot and Q–Q plot.

To study the possible confounding effect of demographic variables on the results, the mixed factorial ANOVA model was repeated, including these data as covariables.

Poisson regression was used to test the effect of the intervention on the low/high risk of Weekly DDQ adjusting for baseline characteristics and unadjusted. The variable number of weekly drinks assessed using the DDQ instrument was categorized into a nominal qualitative variable (high-risk and low-risk consumption) following the indications of the NIAAA that considers low-risk consumption for women no more than seven drinks per week and for men no more than 14 (21). The robust error variance was estimated using the Huber-White (Robust) Sandwich Estimator.

All the analyses were carried out in Stata version 16. For all tests, results for which *p* < 0.05 were considered statistically significant.

### 2.10. Ethical considerations

This research was approved by the Research Ethics Committee of the university where the research was conducted (code: 2021.162). Institutional permission was obtained, and all participants signed a written informed consent form. To maintain confidentiality, the participants' details were codified.

## 3. Results

### 3.1. Participants

**Figure 1:**
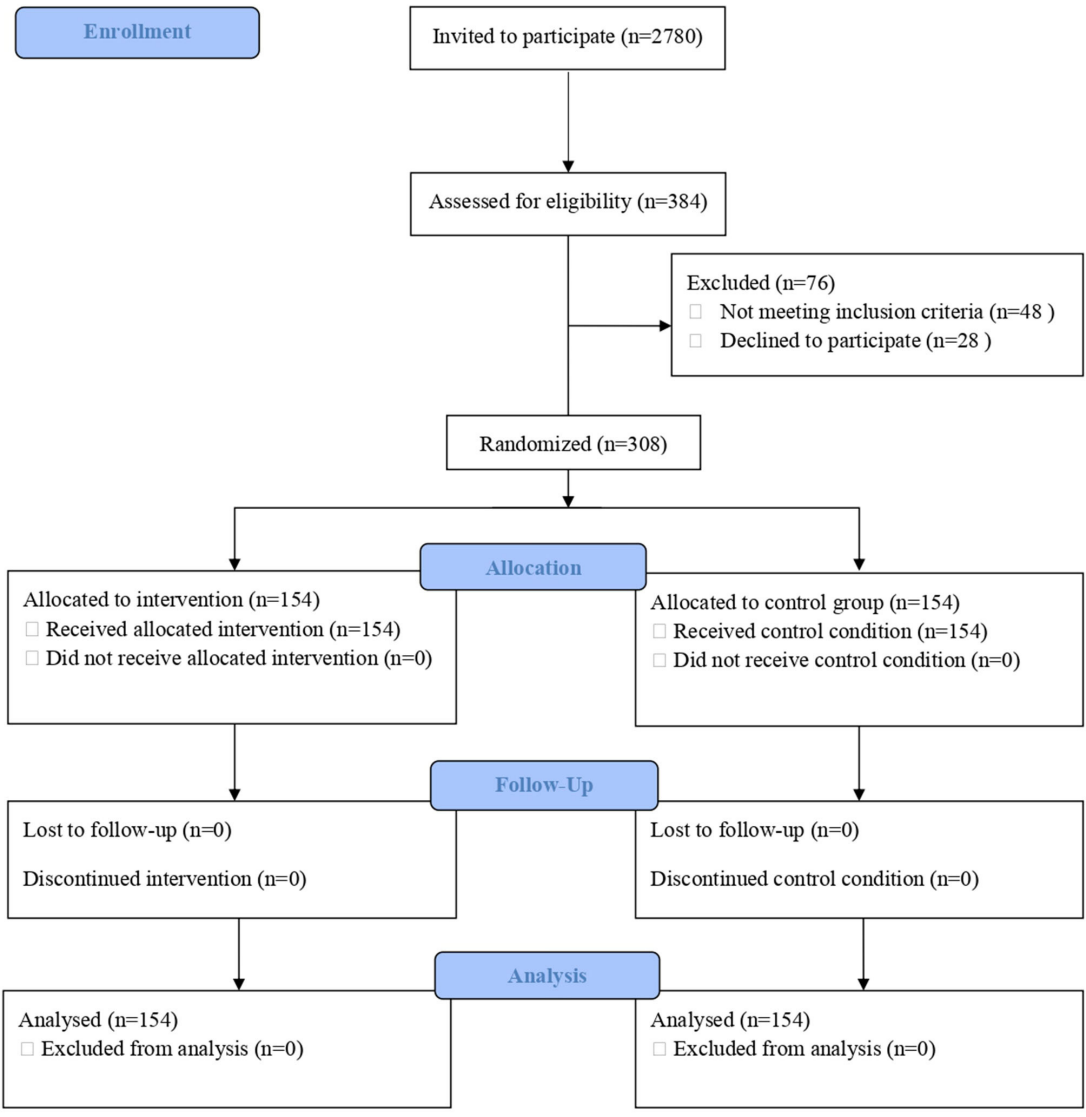
Consolidated Standards of Reporting Trials (CONSORT) flowchart.

Figure 1 shows the flow chart of study participants. A total of 2,780 students were invited to take part in the study, and 384 students agreed to participate. Of them, 48 did not meet the inclusion criteria of having binge drinking episodes; five refused to participate; and 23 could not be contacted by e-mail. A total of 308 students (age range = 18–19 years) met the inclusion criteria and were randomized to the intervention (*n* = 154) or control (*n* = 154) group. Within the intervention group, all subjects completed the intervention protocol. Of the 308 subjects enrolled in the trial, all (100%) completed the 1-month follow-up.

### 3.2. Baseline characteristics

Participants characteristics of the study are shown in Table 1 by treatment condition. The mean age of the students was 18.75 (SD: 0.04) years, and most participants were female (67.5%). Regarding participant residence, 33.4% of participants resided in their family home; 43.2% lived in a student residence or at a college; and 23.4% lived in a student flat. With regard to the student faculty, 40.9% of the participants belonged to a bio sanitary science faculty, 48.0% to a social science faculty, and 11.0% to engineering and architecture. Concerning the student's academic year, 60.7% of the participants were first-year students, and 39.3% were second-year students.

**Table 1:** Baseline characteristics of participants by groups.

**Basal characteristics**	**Control group (*n* = 154)**	**Intervention group** **(*n* = 154)**	***P*-value**
**Demographic characteristics**			
Age, mean (95 % CI)	18.7 (18.6, 18.8)	18.8 (18.6, 18.9)	0.698
Weight, mean (95 % CI)	63.4 (61.6, 65.1)	63.8 (62.0, 65.6)	0.733
Sex, *n* (%)			0.715
Male	52 (33.8%)	48 (31.2%)	
Female	102 (66.2%)	106 (68.8%)	
Hometown, *n* (%)			0.172
Spanish	110 (71.4%)	121 (78.6%)	
South American	26 (16.9%)	17 (11.0%)	
North American	12 (7.8%)	8 (5.2%)	
Central America	2 (1.3%)	5 (3.2%)	
Others (Italian, Hungarian)	4 (2.6%)	3 (2.0%)	
Residence, *n* (%)			0.743
Family home	49 (31.8%)	54 (35.1%)	
Student residence	67 (43.5%)	67 (43.5%)	
Student flat	38 (24.7%)	33 (21.4%)	
Faculty, *n* (%)			0.912
Bio sanitary sciences	60 (39.0%)	61 (39.6%)	
Social sciences	78 (50.6%)	75 (48.7%)	
Engineering and architecture	16 (10.4%)	18 (11.7%)	
Academic year, *n* (%)			0.242
First year	90 (58.4%)	97 (63.0%)	
Second year	64 (41.6%)	57 (37.0%)	
Practice sport, *n* (%)			0.400
No	56 (36.4%)	49 (31.8%)	
Yes	98 (63.6%)	105 (68.2%)	
Age start drinking, mean (95 % CI)	15.5 (15.2, 15.7)	15.4 (15.2, 15.6)	0.460
**Drinking variables, mean (95 % CI)**			
Drinks per week	12.4 (11.0, 13.8)	12.6 (11.2, 14.0)	0.829
Drinks per weekend	10.3 (9.2, 11.5)	10.4 (9.2, 11.6)	0.778
Drinks on peak occasion	11.9 (10.9, 13.0)	12.2 (11.2, 13.2)	0.703
Binge drinking episodes	2.2 (2.0, 2.5)	2.1 (1.9, 2.4)	0.580
Peak BAC on a typical week	0.14 (0.13, 0.16)	0.14 (0.13, 0.16)	0.892
Peak BAC on peak occasion	0.22 (0.21, 0.24)	0.23 (0.21, 0.25)	0.734
Alcohol-related consequences	10.2 (9.1, 11.3)	10.1 (9.0, 11.2)	0.922
Motivation to change alcohol use	3.1 (2.6, 3.5)	2.9 (2.5, 3.3)	0.639
Self-Efficacy	8.0 (7.7, 8.3)	7.8 (7.5, 8.1)	0.355

Pearson's χ^2^ test for qualitative variables. Student's t-test for quantitative variables. BAC, blood alcohol concentration.

There were no significant differences at baseline between the intervention group and the control group for sociodemographic variables and alcohol use and alcohol-related consequence outcomes.

### 3.3. Primary outcomes

A significant intervention effect was observed on the primary outcome alcohol consumption in a typical week (mean: intervention 6.6 vs. control 12.3; *p* < 0.001). Participants in the intervention group significantly reduced the number of drinks by 5.7 (95% CI 5.54, 5.86) compared to the control group. In relation to the epidemiological data, the risk of the intervention group having high-risk alcohol consumption after the intervention was 0.56 times (adjusted for all baseline variables) that of the control group; that is, the risk was reduced by half. The number needed to treat (NNT) was 5 (95% CI 3, 9); therefore, it is necessary to interview five university students for the status of one student to change from high-risk drinking to low-risk drinking.

### 3.4. Secondary outcomes

Table 2 shows the effect on the evaluated outcomes. As observed, all the variables except self-efficacy showed statistically significant changes in favor of the intervention group compared to the control group. The intervention group significantly reduced: (i) the number of drinks consumed in a typical weekend by 5.2 (95% CI 5.07, 5.33); (ii) the number of drinks consumed on the occasion of greatest consumption by 4.9 (95% CI 4.78, 5.02); and (iii) binge drinking episodes by 1.4 (95% CI 1.37, 1.43) compared to the control group. Furthermore, the (iv) peak BAC on a typical week and (v) on the occasion of greatest consumption decreased by 0.06 (95% CI 0.058, 0.062) and 0.09 (95% CI 0.088, 0.092), respectively, in favor of the intervention group. Participants in the intervention group also significantly reduced the (vi) number of alcohol-related consequences by 5.8 (95% CI 5.67, 5.93). In addition, they increased (vii) their motivation to change their alcohol use by−0.8 (95% CI−0.85,−0.75). Finally, no statistically significant results were found in relation to (viii) self-efficacy.

**Table 2:** Comparative data for the effect of intervention on change in the evaluated outcomes at one month follow up.

	**Control Group (*n*** = **154)**	**Intervention Group (*n*** = **154)**	***P*-value** ^*^	**Difference (95% CI)**
Drinks per week	12.3 (11.0, 13.7)	6.6 (5.2, 7.9)	< 0.001	5.7 (5.5–5.9)
Drinks per weekend	10.4 (9.4, 11.8)	5.2 (4.1, 6.4)	0.001	5.2 (5.1, 5.3)
Drinks on peak occasion	11.8 (10.8, 12.9)	6.9 (5.9, 7.9)	< 0.001	4.9 (4.8, 5.0)
Binge drinking episode	2.3 (2.0, 2.6)	0.9 (0.6, 1.2)	< 0.001	1.4 (1.4, 1.4)
Peak BAC on a typical week	0.14 (0.13, 0.16)	0.08 (0.06, 0.09)	< 0.001	0.6 (0.5, 0.7)
Peak BAC on peak occasion	0.22 (0.21, 0.24)	0.13 (0.11, 0.15)	< 0.001	0.9 (0.8, 1.0)
Alcohol-related consequences	10.2 (9.1, 11.3)	4.4 (3.3, 5.5)	< 0.001	5.8 (5.6, 5.9)
Motivation to change alcohol use	3.2 (2.7, 3.6)	4.0 (3.5, 4.4)	< 0.001	−0.8 (−0.8, −0.7)
Self-efficacy	7.9 (7.6, 8.2)	8.1 (7.9, 8.5)	0.274	−0.2 (−0.2, −0.1)

^*^P-value from Student's t-test. BAC, blood alcohol concentration.

## 4. Discussion

This is the first study to evaluate the effectiveness of the peer-led BASICS intervention in reducing alcohol consumption among Spanish university students. The results confirm our main hypothesis, indicating that the intervention positively impacts alcohol use by significantly decreasing the number of drinks consumed in a typical week. In addition, the findings in the secondary outcomes were promising, demonstrating significant reductions in the drinks per weekend, drinks on peak occasion, binge drinking episodes, peak BAC on a typical week, peak BAC on peak occasion, alcohol-related consequences and the motivation to change alcohol use. These findings are in line with a recent meta-analysis that showed that BASICS was the most effective intervention among brief alcohol interventions delivered in the college setting (7). The only variable that did not change significantly was self-efficacy.

Our main finding was that participants in the intervention group significantly reduced the number of drinks per week by 5.7 compared to the control group. In line with our results, only three studies including the peer-led BASICS intervention have shown a significant reduction in the number of drinks per week by 5.24 (22), 0.89 (23), and 2.5 in students who receive 50-min BASICS intervention and 4.1 in the 10-min intervention group (24).

In addition, to reinforce the encouraging results related to the main hypothesis, our study is the first investigation demonstrating the impact of peer-led BASIC intervention on drinks per weekend, drinks on peak occasion, peak BAC on a typical week, and the motivation to change alcohol use. Regarding the number of binge drinking episodes, our results suggest that the intervention exceeds the results of previous studies. While our study revealed a reduction of 1.4 episodes of binge drinking, Pueyo-Garrigues et al. (13) found a reduction of 0.05. Concerning the peak BAC on the occasion of greatest consumption, our results are in line with those found in the literature, since we found that the undergraduates reduced their peak BAC by 0.09, which is similar to the findings of (13). However, Turrisi et al. (23) achieved a smaller reduction of 0.015. Finally, no previous peer-led BASICS study has been shown to reduce alcohol-related consequences (10); however, in our study, a reduction of 5.8 in the number of consequences was achieved.

There could be several explanations for these encouraging findings. First, BASICS intervention has been shown to be effective in addressing alcohol use in college students (25) because it follows the harm reduction philosophy and aims to reduce the potential and real risks derived from alcohol intake rather than seeking abstinence (6, 26). Young people are known to respond best to this type of alcohol prevention approach (26). Moreover, the intervention combines the motivational intervention with personalized feedback. Addressing each student's motivation is key, as it has been found to be a strong predictor of drinking behavior in short- and long-term outcomes (27). The personalized feedback sheet is also essential, as giving individualized data on alcohol use has been shown to reduce short-term drinking frequency and symptom severity (28).

Given the demonstrated effectiveness of BASICS, we believe that the size of the effect obtained is also due to the fact that the intervention was led by peers who have received specific training. This can be a strength since the values, habits and lifestyles of young people are especially susceptible to peer influence (29). Peer relationships play a central role in the lives of young people, and the effects of peer influence are stronger during this period than in adulthood (30). It is known that students learn more effectively from their peers than individuals in other generations (31). In fact, students value their peers as organized and well-prepared, knowledgeable about the subject, and good transmitters of information, which makes them feel comfortable and nonjudgmental (12, 31). Therefore, at this age, the influence of peers on health and wellbeing is greater than at any other age (32), making the use of peer-to-peer approaches in youth-targeted health interventions a promising health promotion strategy.

Additionally, the training of the peer counselors who have led the BASICS intervention might be another key element related to the success of this study. Students received a training program with proven effectiveness in conducting motivational interviews about alcohol consumption with their peers (33). After receiving the training, participants reported that they obtained the necessary tools to conduct motivational interviewing and that they felt more confident and credible in addressing alcohol use with their peers (33). In addition, these peer counselors were nursing students who are characterized by having a greater development of empathy, better communication skills, and an increased ability to understand the needs of the person, develop appropriate responses, and individualize care plans (34–36). These aspects could have positively impacted the effectiveness of the intervention. Spanish university students can benefit from this intervention since by reducing alcohol consumption they will experience individual short-term benefits such as better sleep quality, more energy, greater money savings, improved memory, reduced risk of alcohol poisoning and alcohol dependence, improvements in mental health and academic performance; and better overall physical and mental health (37–39). Furthermore, it has also social advantages such as the decrease in the rates of traffic accidents, violence, and a reduction in alcohol-related health expenditure (37, 40). Additionally, reducing binge drinking episodes is associated with a better quality of life, especially greater vitality and mental health (41). Moreover, undergraduates typically do not utilize health services and are not usually accessible by health professionals (42, 43). This aspect is crucial since undergraduates are in a critical period for the development and consolidation of their future lifestyles (44). Although university authorities and public policy makers have attempted to address elevated levels of consumption, research points to an increase in alcohol use among students in the last decade (45). However, this intervention led by university students has shown that it can positively impact alcohol consumption in this population with approximately half of the students in the intervention group changing their consumption pattern from high risk to low risk. In addition, previous research shows that this type of intervention not only impacts the student who receives it but also has a positive effect on the counselor who leads it (33). In addition, the observation that, for every five university students who receive the intervention, one goes from high-risk to low-risk consumption is a very promising finding as this suggests the intervention is feasible given that few interventions are demonstrated to be effective.

Three main limitations in this study need to be recognized. First, the study was carried out at a single university, so the participants in the treatment groups could have influenced each other. However, the importance of not sharing the information received to participants in the control group was impressed upon participants in the intervention group and an intention-to-treat analysis was applied to avoid the effects of crossover. Second, as we aimed to determine the short-term effects of the intervention, the follow-up period was short. For future studies, it would be interesting to carry out measurements over an extended follow-up period to determine the long-term effects of the intervention. Third, the outcome measures were all self-reported. To try to avoid any bias in these measurements, participants were insisted that they were not going to be judged and that they should respond as honestly as possible. In addition, we follow recommendations to improve the reliability and validity of these measures, such as: collecting data online, asking for longer timeframes and questions which involve specified timeframes; and, including beverage-specific questions (46).

Despite these limitations, this study had some strengths. First, all the participants assigned to the intervention group received the motivational interview. Furthermore, no participants in either group were lost to follow-up. This may be due to the commitment that the students acquired by participating in the study, their positive experience when receiving the interview, the relationship established with the peer counselors, or the reminders sent through WhatsApp. Second, the students who led the intervention were totally devoted to the project, and after each interview, peer facilitators spoke with the research team to discuss how the interview had gone, their concerns and areas for improvement. Finally, the selected intervention is underpinned by a strong foundation in behavior change theory, peer-led interventions, motivational interviewing principles, and harm reduction approaches, which have been shown to be appropriate for this age group.

## 5. Conclusion

In conclusion, the peer-led BASICS intervention was effective in changing alcohol consumption among Spanish university students in the short term. Nursing students positively impacted the drinking pattern of their peers, achieving significant decreases in the amount of alcohol consumption, binge drinking episodes, peak BAC, and alcohol-related consequences. Nursing undergraduates can be considered a great asset for health promotion at the university setting. Future studies are needed to determine the long-term effectiveness of peer-led BASICS interventions.

## Data availability statement

The raw data supporting the conclusions of this article will be made available by the authors, without undue reservation.

## Ethics statement

The study involving humans was approved by Research committee from the University of Navarra. The study was conducted in accordance with the local legislation and institutional requirements. The participants provided their written informed consent to participate in this study.

## Author contributions

ML-G: Conceptualization, Formal analysis, Funding acquisition, Investigation, Methodology, Writing—original draft. MP-G: Conceptualization, Funding acquisition, Methodology, Supervision, Writing—review & editing. DC: Formal analysis, Writing—review & editing. NE-L: Writing—review & editing. CA-D: Writing—review & editing. NC-A: Conceptualization, Funding acquisition, Methodology, Supervision, Writing—review & editing.
